# Life History Trade-Offs and Relaxed Selection Can Decrease Bacterial Virulence in Environmental Reservoirs

**DOI:** 10.1371/journal.pone.0043801

**Published:** 2012-08-24

**Authors:** Lauri Mikonranta, Ville-Petri Friman, Jouni Laakso

**Affiliations:** 1 Centre of Excellence in Biological Interactions, Department of Biological and Environmental Science, University of Jyväskylä, Jyväskylä, Finland; 2 Department of Biological and Environmental Science, University of Helsinki, Helsinki, Finland; 3 Department of Zoology, Oxford University, Oxford, United Kingdom; Virginia Tech, United States of America

## Abstract

Pathogen virulence is usually thought to evolve in reciprocal selection with the host. While this might be true for obligate pathogens, the life histories of opportunistic pathogens typically alternate between within-host and outside-host environments during the infection-transmission cycle. As a result, opportunistic pathogens are likely to experience conflicting selection pressures across different environments, and this could affect their virulence through life-history trait correlations. We studied these correlations experimentally by exposing an opportunistic bacterial pathogen *Serratia marcescens* to its natural protist predator *Tetrahymena thermophila* for 13 weeks, after which we measured changes in bacterial traits related to both anti-predator defence and virulence. We found that anti-predator adaptation (producing predator-resistant biofilm) caused a correlative attenuation in virulence. Even though the direct mechanism was not found, reduction in virulence was most clearly connected to a predator-driven loss of a red bacterial pigment, prodigiosin. Moreover, life-history trait evolution was more divergent among replicate populations in the absence of predation, leading also to lowered virulence in some of the ‘predator absent’ selection lines. Together these findings suggest that the virulence of non-obligatory, opportunistic bacterial pathogens can decrease in environmental reservoirs through life history trade-offs, or random accumulation of mutations that impair virulence traits under relaxed selection.

## Introduction

Pathogen virulence (measured as the severity of a disease) is often assumed to evolve in a strict co-evolutionary arms race between the pathogen and its host [1]–[5]. The theory of virulence also commonly assumes that pathogen reproduction, and consequently the evolution of virulence, is entirely dependent on the host species [2]. While this view might hold for obligate pathogens, it seems inaccurate for opportunists that are capable of reproducing outside their hosts [6]–[7]. Previous studies have shown that opportunists are exposed to many different selective pressures in environmental reservoirs, which could have correlative effects on bacterial virulence (‘coincidental selection hypothesis’) [8]–[14]. For example, toxicity and tolerance against degradative enzymes of mammalian macrophages may have evolved originally as defence mechanisms against protist predation [12], [15]–[17]. In addition to this “dual-use” of virulence factors, pathogenicity could be, for example, an evolutionary remnant of adaptation for accidental passage through another organism, or merely an inevitable consequence of within-host persistence in microbes that hitch-hike in their hosts to disperse into new locations [6]. Even so, virulence evolution in opportunistic pathogens is usually not considered in a wider ecological context across different environments [7].

Even though virulence and survival in the outside-host environment correlate positively in some pathogens, environmental bacterial isolates are seldom as virulent as clinical isolates of the same species [18]–[19]. Therefore, it is possible that selection in the outside-host environment conflicts with bacterial pathogenicity: traits needed for survival are traded off with traits connected to virulence so that an investment in one trait leads to a corresponding decrease in another. This ‘conflicting selection hypothesis’ is seldom directly tested but some studies support it demonstrating that virulence traits can incur fitness costs in environmental reservoirs [8], [20]–[22]. Fitness trade-off between within-host and outside-host environments has also been observed in some [23] but not all plant pathogens [24]. Instead of trading off with survival traits, virulence traits could also be neutral in the external environment. In that case, virulence traits could be lost even without negative selection if they are impaired due to random accumulation of mutations (‘relaxed selection hypothesis’) [25]–[26].

Here we tested the ‘conflicting selection’ and ‘relaxed selection’ hypotheses by studying how predation by *Tetrahymena thermophila* protist changes the defensive and virulence traits of an opportunistic bacterial pathogen, *Serratia marcescens*. We define an opportunistic bacterium as a pathogen, which does not require a host for reproduction, and which can be transmitted between hosts through the environment [7]. *S. marcescens* is a prime example of such an opportunist: it is able to infect a wide range of plant, invertebrate and vertebrate hosts (including humans) and is commonly found in different environmental reservoirs [27]–[29]. Previous experiment showed that protozoan predation decreases *S. marcescens* virulence in *Parasemia plantaginis* lepidopteran host, when genetically diverse inocula (i.e. mixture of numerous clones with different characteristics) are used for infections. The experiment suggested that the decrease in virulence was connected to reduced growth rate and motility, and to loss of a red pigment, prodigiosin [13].

Here we study the changes in these traits (pigmentation, biofilm, growth rate and motility) in more detail by using single bacterial clones that have evolved in the absence or presence of a protist predator in a long-term selection experiment. First, we investigate whether we can link changes in these traits into either virulence or anti-predator defence. Second, we examine whether the previously observed pattern of decreased virulence emerges when intraspecific interactions that arise due to diversity (e.g. competition, cooperation and cheating [30]–[34]) are excluded during infection. For example, competition between different bacterial genotypes can affect the severity of infection [33]–[35] but the use of single clones should exclude competition. Third, we study whether changes in bacterial life-history traits are similar (parallel evolution) or different (divergent evolution) among the replicate populations within the ‘predator present’ and ‘predator absent’ conditions: if replicate selection lines diverge more clearly in the absence of predation, it would indicate relaxed selection in an enemy-free environment.

Our results suggest that when the natural protist enemy is present, survival of *S. marcescens* can correlate negatively with virulence (conflicting selection). At the same time, however, most life history traits (including virulence) are likely to diverge more between replicate selection lines in the absence of a strong selective agent such as predation (relaxed selection).

## Materials and Methods

### The long-term evolutionary experiment and the isolation of clones

We used clones isolated from a prior long-term experiment, where the bacterium (a single ancestral clone from ATCC#13880 strain of *Serratia marcescens* ssp. *marcescens*) was exposed to a predatory protist, *Tetrahymena thermophila* (ATCC #30008), for 13 weeks totalling approximately 1300 bacterial generations (for a detailed description, see: [36]). We used four populations that had evolved in the presence or absence of protists, and randomly isolated eight clones per population (a total of 64 clones, 32 clones per treatment). All populations and the ancestral clone stored in −80°C were first thawed, diluted and plated on agar plates (10 g of Difco™ nutrient broth, 2.5 g of Bacto™ yeast extract and 15 g of Bacto™ agar in 1 L of dH_2_O). After 48 h of cultivating at 25°C, individual clones were randomly picked and cryopreserved separately in −80°C (mixed with 45% of glycerol and 9% of Nutrient Broth: 10 g of Difco™ nutrient broth, 2.5 g of Bacto™ yeast extract in 1 L of dH_2_O. Becton, Dickinson and Co., Franklin Lakes, NJ). We recorded the colony colour of every isolated clone (red or white indicating prodigiosin pigment synthesis or lack thereof, respectively). The colony colour frequencies in the populations were also recorded.

### Measuring changes in bacterial life history traits

We measured bacterial ability to sustain biomass in the presence of predators (i.e. defence), ability to form biofilm (cell aggregates attached to surfaces) and maximum growth rate in liquid culture medium with Bioscreen C™ spectrophotometer (optical density measured with wideband option: 420–580 nm, 25°C, 400 µL volume; Growth Curves Ltd, Helsinki, Finland). While optical density (OD) is not an exact measure of bacterial cell numbers, it can be used for reliably comparing differences in bacterial growth [13], [37]. We cultivated the bacteria in cereal leaf extract medium, which was also used in the prior long-term experiment [36]: 1 g/L of leaf extract (Cerophyll™, Ward's natural science) was first boiled for 5 min in dH_2_O, cooled down and filtered through glass fibre filter (CF/C, Whatman) resulting in a final concentration of 2.15 mg plant detritus/L. After autoclaving (121°C, 20 min.), the medium was adjusted to pH 7.5 with sterile phosphate buffer (K_2_HPO_4_·3H_2_O 1.5724 g, KH_2_PO_4_ 0.4 g, (NH_4_)2SO_4_ 0.5 g, MgSO_4_·7H_2_O 0.1 g, NaCl 0.01 g and CaCl_2_·2H_2_O 0.0228 g in 1 L of dH_2_O).

Bacterial defence and ability to form biofilm in the presence of predators were measured as follows. The clones were grown individually to similar high densities on microplates (in 370 µL of fresh culture medium; Honeycomb 2 microtitre plates, Thermo Electron Oy, Vantaa, Finland) before adding 30 µL inoculum of protist predators (approximately 100 *T. thermophila* individuals), which then reduced the bacterial biomass by grazing. Defence was measured as bacterial biomass after 93 h of cocultivation with the predator: the higher the OD, the better the defence.

Biofilm formation was measured as follows: 100 µL of 1% crystal violet solution (Sigma-Aldrich) was added into the microplate wells and rinsed off with distilled water after 10 minutes. The remaining crystal violet attached to bacteria was dissolved in 96% ethanol, and the amount of biofilm formed was measured as OD at 420–580 nm (a method modified from [38]).

To measure maximum growth rate (r_max_) we introduced a 10 µL inoculum of bacteria into 400 µL of fresh culture medium. Growth was measured as the change in OD in five-minute intervals as described above.

Motility was assessed by sticking a trace inoculum (∼2 µL) of each clone onto the centre of a semi-fluid agar plate (as described above, except 0.7% agar) with a sterile loop (VWR). The plates were photographed after 48 h, and the colonised area was determined with ImagePro Plus 4.5 software (Media Cybernetics).

### Measuring bacterial virulence

Virulence was measured using wax moth larvae (*Galleria mellonella*, Lepidoptera; Pyralidae) as hosts. Bacterial virulence measured in *G. mellonella* correlates with virulence measured in mammals and mammalian cell cultures, making the larvae an ideal model host for general virulence testing [39]–[41]. Our larvae were randomly selected from four different batches (Kreca V.O.F, Ermelo, Netherlands). We used each of the 64 bacterial clones to infect ten larvae: a total of 320 individuals were infected with ‘predator present’ clones and 320 individuals with ‘predator absent’ clones. We also infected 60 larvae with the ancestral clone. Additionally, 40 larvae were injected with distilled water to control for the damage caused by the injection itself (total n = 740 larvae). The larvae in these four treatment groups had comparable mean body mass (ANOVA, F_3, 796_ = 0.022, p = 0.996, water: M = 121.9, SD = 42.4, predator present: M = 120.8, SD = 34.1, predator absent: M = 119.2, SD = 29.8, ancestor: M = 123.1, SD = 41.2

The bacterial clones were first thawed and spread on agar plates at high density. After 48 hours of incubation at 25°C, the bacterial mass was collected by scraping, mixed with phosphate buffer (described above in the context of cereal leaf extract medium) and diluted to OD 2.0 at 420–580 nm. Before infection, all clones were further diluted with the phosphate buffer into density of 16.6×10^8^ CFU ml^−1^ (+/−1.6×10^8^ CFU mL^−1^). Bacterial densities (in CFU) did not differ between the treatments (ANOVA, F_2, 69_ = 0.829, p = 0.441). The larvae were injected between the abdominal segments six and seven with 5 µL (on average 8.3×10^6^ CFU) of the solution using a Hamilton syringe. Infected larvae were placed individually on empty Petri dishes and their survival was monitored at three to twelve-hour intervals for six days at 25°C. The larvae were infected during four consecutive days in constant conditions. Injection day had no effect on survival (Kaplan-Meyer survival analysis, log-rank statistics, χ^2^ = 0.349, p = 0.951), and all the treatments were injected in random order.

### Statistical analyses

We used ANOVA (GLM) to explain variance in the dependent variables (life-history traits) with predation treatment (predator absent or predator present) and colony colour (red or white) as fixed factors, including predation×colony colour interaction. The effect of population identity was taken into account in the main analysis by nesting replicate selection lines within the predation treatments as random factors. Population divergence was further studied within both predation treatments separately by fitting the replicate selection line as a random factor into the model; significant population effect indicates divergence within the treatment with respect to the given trait [42]. Five outlier data points were excluded from motility analysis because of swarming behaviour [43].

Bacterial virulence was analysed as host survival with Kaplan-Meier survival analysis and log-rank statistics. For the ANOVA (see above) and for a genetic correlation analysis virulence was also compressed to a mean virulence value (per clone) by taking the inverse of the average time (h) that was required for a given clone to kill the host replicates (*Virulence* = 1/[mean time of death]). All the survived larvae were given a maximum survival value of 150 h.

Genetic correlations were first analysed at the level of bivariate correlations (Pearson's correlation coefficient between two traits measured from individual clones) within predation treatments (present or absent) by pooling clones together regardless of their population origin. These results were then contrasted with an analysis of covariance by (1) including the effect of population identity as a cofactor in the linear regression model (one trait explained with another) and (2), including both population identity and colony colour as cofactors.

Statistical analyses were performed with SPSS-software (v.20.0, SPSS Inc., Chicago, IL).

## Results

### Evolutionary changes in bacterial colony colour frequencies and life-history traits

Protist predation decreased the frequency of prodigiosin pigment-synthesizing clones in the experimental populations (F_1, 6_ = 43.7, p = 0.001): only 32.4% of all the bacterial colonies were red after evolving in the presence of predators compared to 89.1% in the absence of predators.

**Figure 1:**
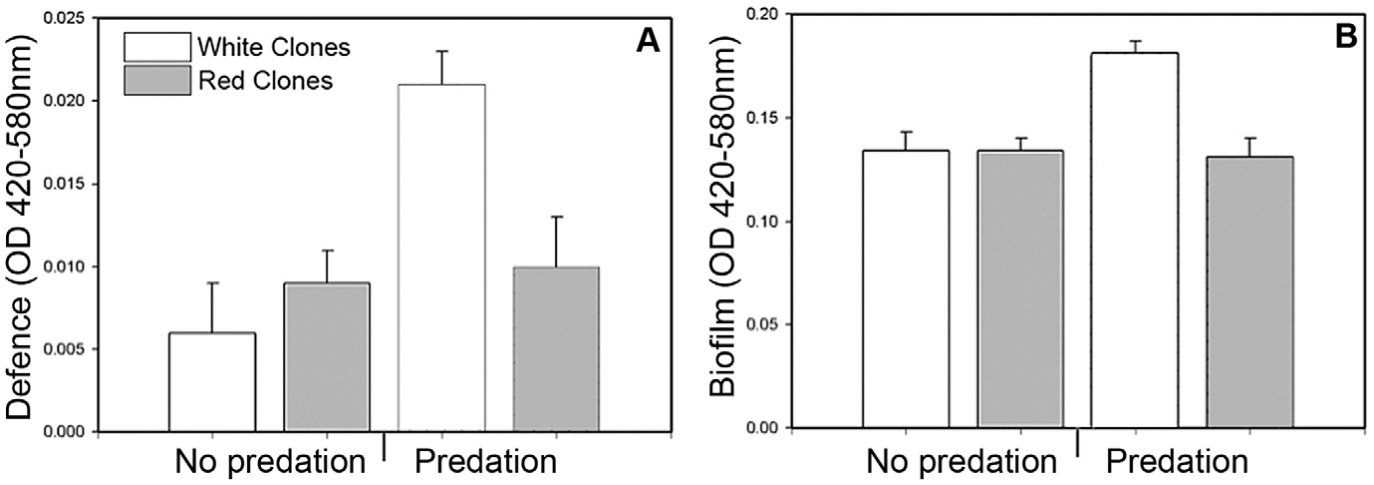
Effect of predation on bacterial defence and biofilm formation. (A) The bacterial clones' defence, i.e. ability to sustain biomass, and (B) ability to form biofilm in the presence of predators within the ‘predator present’ and ‘predator absent’ treatments. White bars denote white, and grey bars denote red bacterial clones. Error bars denote 2 s.e.m.

White bacterial clones that had evolved in the presence of protists during the previous long-term selection experiment were able to sustain higher biomass in the presence of these predators than white clones that had evolved with the protists absent. However, the biomass of red clones did not depend on predation treatment. In other words, the effect of previous predation on the evolution of bacterial defence interacted with prodigiosin synthesis (Fig. 1A & Table 1). Similar interaction was also found in the formation of predation-resistant biofilm: white clones formed more biofilm than the red only if they had evolved in the presence of predators (Fig. 1B & Table 1). Consequently, the bacterium's ability to sustain biomass and form biofilm in the presence of predators correlated positively (Pearson's r = 0.875, p<0.001) due to the emergence of highly defensive white clones. Predation treatment, colony colour, or their interaction had no effect on bacterial maximum growth rate. Motility was only affected by the colony colour: red clones were more motile than white clones (Table 1).

**Table 1:** Effects of predation, pigment synthesis, predation×pigment synthesis and replicate population on trait means.

	Predation:			Prodigiosin synthesis:			Predation×Prodigiosin:		Pop^1^, Predator absent:		Pop^1^, Predator present:	
	test values	significance	direction^2^	test values	significance	direction^2^	test values	significance	test values	significance	test values	significance
**Defence**	F_1, 6.4_ = 8.1	**p = 0.027**	increase	F_1, 52_ = 2.4	p = 0.130	0	F_1, 52_ = 11.5,	**p = 0.001**	F_3, 27_ = 3.4,	**p = 0.032**	F_3, 27_ = 1.6	p = 0.203
**Biofilm**	F_1, 6.7_ = 4.9	p = 0.064	0	F_1, 54_ = 10.7	p = 0.002	decrease	F_1, 5_4 = 10.3	**p = 0.002**	F_3, 28_ = 0.1	p = 0.940	F_3, 28_ = 1.0	p = 0.389
**rmax**	F_1, 6.7_ = 0.2	p = 0.696	0	F_1, 54_ = 0.5	p = 0.447	0	F_1, 54_ = 1.2	p = 0.272	F_3, 28_ = 85.0	**p<0.001**	F_3, 28_ = 35.7	**p<0.001**
**Motility**	F_1, 6.2_ = 0.1	p = 0.768	0	F_1, 49_ = 5.4	p = 0.025	increase	F_1, 49_ = 0.3	p = 0.573	F_3, 27_ = 14.1	**p<0.001**	F_1, 49_ = 0.3	p = 0.573
**Virulence**	chi^2^ = 10.7	**p = 0.001**	decrease	chi^2^ = 46.1	**p<0.001**	increase	**Table S2**	**Table S2**	F_3, 31_ = 3.9	**p = 0.018**	F_3, 28_ = 1.22	p = 0.318

1 Pop describes the effect of replicate selection lines within the treatments (predator absent or predator present) on the trait means, i.e. the divergence of populations with identical selective environment.

2 The direction (where applicable) means the direction of mean change of the ‘predator present’ treatment compared to the ‘predator absent’ treatment, or the direction of mean change of the red clones compared to the white clones. Null (0) denotes no difference between these treatments.

Absence of predators led to more divergence between the replicate selection lines. Populations in the ‘predator absent’ treatment diverged in all measured traits except the formation of predation-resistant biofilm (defence, growth rate, motility and virulence, Table 1). Replicate populations within the ‘predator present’ treatment differed only in their maximum growth rate (Table 1).

### Evolutionary changes in virulence

**Figure 2:**
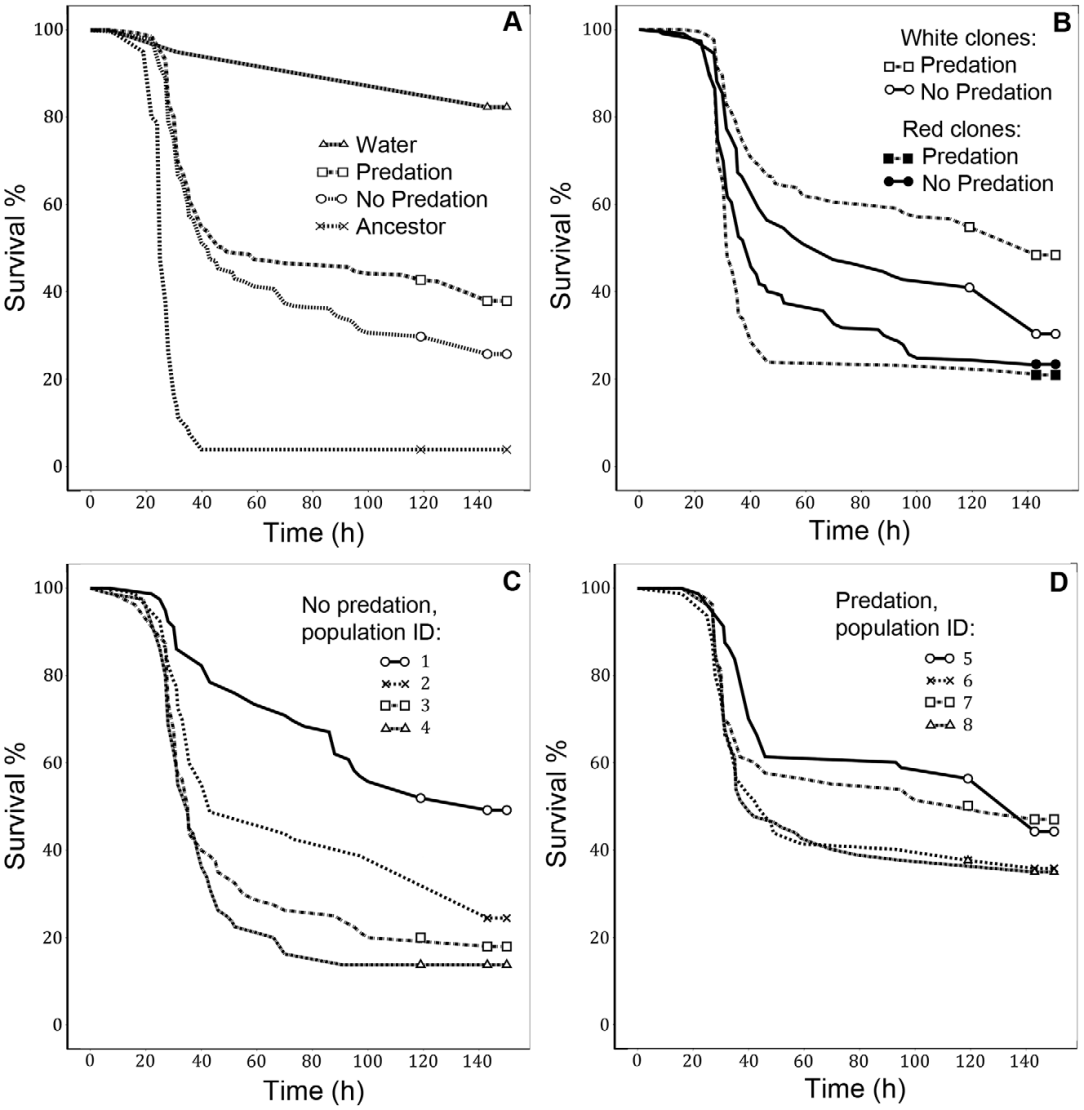
Survival of *G. mellonella* larvae when infected with different bacterial clones. Larvae infected with (A) clones from the ‘predator present’ and ‘predator absent’ treatments, the ancestral clone, and the water control, with (B) white and red clones from the ‘predator present’ and ‘predator absent’ treatments, and with (C & D) the clones from different replicate populations of the ‘predator present’ and ‘predator absent’ treatments.

Protist predation clearly reduced bacterial virulence, whereas the clones that had evolved in the absence of predators were intermediately virulent compared to the most virulent ancestral strain (Fig. 2A, main effect of selection line: χ^2^ = 245.66, p<0.001. For pairwise comparisons including the water control treatment, see Table S1). Red clones were generally more virulent than white ones within both predation treatments (χ^2^ = 46.120, p<0.001, Table 1). White clones that had evolved in the presence of predators were the least virulent (pairwise comparison for the effect of colony colour within predation treatment: χ^2^ = 6.80, p = 0.009, Fig. 2B, Table S2).

Replicate selection lines diverged with respect to their virulence only within the ‘predator absent’ treatment (Table 1, in Fig. 2C&D).

### Life-history trait correlations

**Figure 3:**
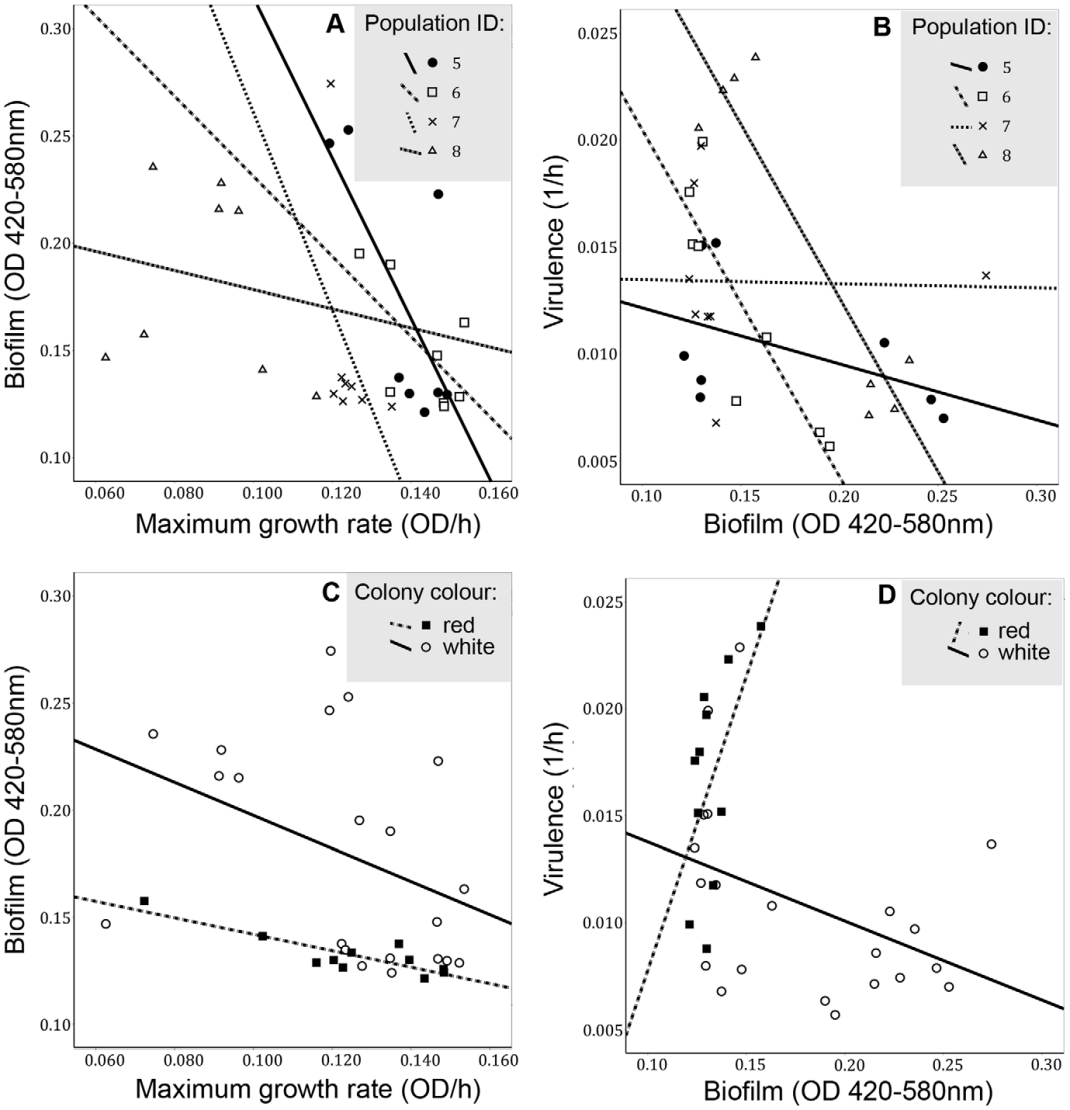
Bacterial life-history trait correlations within predation treatments. Population-level (replicate selection lines numbered 5–8) correlations between (A) predation-resistant biofilm and maximum growth rate, and (B) virulence and predation-resistant biofilm. Correlations between (C) predation-resistant biofilm and maximum growth rate by colony colour, and (D) virulence and predation-resistant biofilm by colony colour.

None of the bivariate correlations or linear regression models (ANCOVA) of life-history traits were significant within the ‘predator absent’ treatment (Table 2). In contrast, two significant correlations were found within the ‘predator present’ treatment: formation of predation-resistant biofilm correlated negatively with both maximum growth rate and virulence (Table 2). Population affected both correlations suggesting that the evolution of biofilm had different effects on maximum growth rate and virulence among replicate selection lines within the predation treatment (Table 2, Fig. 3A&B). However, including population identity in the linear regression model did not turn non-significant covariates significant, or vice versa (Table 2). Colony colour affected only the correlation between virulence and predation-resistant biofilm: adding both population identity and prodigiosin as cofactors into the regression model erased the negative correlation between predation-resistant biofilm and virulence, which suggests that variation in virulence was best explained by differences between the white and the red clones (Table 2, Fig. 3C&D).

**Table 2:** Pairwise correlations and covariance analyses with population and pigment synthesis fitted as cofactors.

Predator absent:	Pearson correlation:		Linear regression with population as a covariate:				Linear regression with population and prodigiosin as covariates:				
	*r*	*P*	*R2*	*P*	*Covariate*	*Pop* 1	*R2*	*P*	*Covariate*	*Pop* 1	*Prod* 2
Virulence-Motility	−0.226	0.222	0.136	0.129	0.912	0.108	0.155	0.202	0.895	0.151	0.45
Virulence-rmax	−0.334	0.062	0.157	0.083	0.954	0.219	0.18	0.129	0.995	0.207	0.382
Virulence-BF	0.007	0.972	0.159	0.081	0.823	**0.026**	0.183	0.125	0.788	**0.029**	0.374
rmax-BF	0.138	0.972	0.69	**0.001**	0.49	**0.001**	0.692	**0.001**	0.51	**0.001**	0.725
Motility-BF	−0.008	0.966	0.427	**0.001**	0.856	**0.001**	0.483	**0.001**	0.849	**0.001**	0.101

Predator present:											
	*r*	*P*	*R2*	*P*	*Covariate*	*Pop* 1	*R2*	*P*	*Covariate*	*Pop* 1	*Prod* 2
Virulence-Motility	0.19	0.333	0.21	0.053	0.085	**0.027**	0.437	**0.003**	0.39	**0.024**	**0.005**
Virulence-rmax	−0.243	0.18	0.116	0.167	0.842	0.182	0.373	**0.004**	0.901	0.223	**0.002**
Virulence-BF	−0.457	**0.009**	0.352	**0.002**	**0.003**	**0.017**	0.446	**0.001**	0.064	**0.018**	**0.037**
rmax-BF	−0.366	**0.039**	0.83	**0.001**	**0.006**	**0.001**	0.843	**0.001**	**0.005**	**0.001**	0.356
Motility-BF	−0.266	0.171	0.172	0.094	0.189	0.093	0.223	0.103	0.685	0.087	0.221

Pairwise (Pearson) correlations of trait pairs, and covariance analyses (linear regression) with replicate selection line, and replicate selection line with colony colour fitted as covariates. The first trait of the trait pairs is the dependent variable in the regression analyses. Significant values (>0.05) are highlighted.

1 Effect of replicate selection line, i.e. population identity.

2 Effect of colony colour, i.e. prodigiosin synthesis.

## Discussion

We studied experimentally how protist selection affects bacterial defensive adaptations and virulence measured *in vivo*. We also studied which life-history traits are connected to anti-predator defence and virulence, and whether these are genetically correlated. Furthermore, we investigated whether evolutionary changes in life-history traits are consistent (parallel evolution) or different (divergent evolution) among replicate populations: more population divergence would indicate relaxed selection.

Our results show that protist predation increased the frequency of non-pigmented (white) *S. marcescens* clones that were more defensive but less virulent compared to the ancestral-like red clones. Bacterial defence, i.e. the ability to sustain high biomass in the presence of a predator, was mechanistically connected to the formation of predation-resistant biofilm, which protects several bacterial species from various protist predators [11], [14], [44]. Adaptation to predation with biofilm formation led to negative correlation with both maximum growth rate and virulence. Reduced competitive ability (i.e. lowered growth rate) can lead to less efficient host exploitation and hence decreased virulence [2], [33] but we did not find correlation between virulence and growth rate: maximum growth rate did not explain variation in virulence (Table 2). Therefore, even though anti-predator defence incurred a clear fitness cost in terms of reduced growth rate (Fig. 3 A, Table 2), it was not directly related to virulence. One explanation for this is that growth rate in a plant-based culture medium is a poor proxy for *S. marcescens'* growth and virulence within an insect host. Contrary to previous findings that biofilm formation correlates positively with both bacterial virulence and anti-predator defence [44], [45], we found a negative genetic correlation between these traits: predator-induced increase in the formation of biofilm decreased virulence (Table 2., Fig. 3).

This decrease in virulence was best explained by the loss of prodigiosin synthesis although clinical *S. marcescens* isolates have been primarily found to be non-pigmented [29]: white clones were consistently less virulent regardless of their defensive ability, whereas red clones were consistently poorly defended and more virulent than white ones (Fig. 1 & 2B). Because prodigiosin is toxic for some eukaryotic cells [45], and prodigiosin expression and virulence can be regulated pleiotropically [46]–[47], it is possible that the loss of pigmentation directly reduced *S. marcescens* virulence in the wax moth host. Alternatively, prodigiosin expression could be tightly linked with some other important virulence factor such as protease production [48].

Motility had no clear effects on virulence (Table 2), even though it has previously been linked to *S. marcescens* virulence [13]–[14] and bacterial pathogenicity in general [49]–[52]. Measuring motility at the level of populations (clone mixes) can be confounded by intraspecific interactions between clones (e.g. competition, cooperation and cheating). It is known that bacterial motility can increase when cells cooperate in producing surfactants that turn the microenvironment more suitable for moving [53]. Bacteria might, however, cooperate less and consequently move less when relatedness of the population decreases, i.e., population becomes more diverse due to the emergence of cheating genotypes [30], [54]. This kind of social conflict could explain why motility was observed to decrease with virulence in a previous experiment where bacterial traits were measured at population level (multiple clones interacting) [13], whereas we found no difference in this experiment because relatedness was the highest possible (motility measured at the level of individual genotypes).

Somewhat surprisingly, virulence decreased also in some of the replicate selection lines within the ‘predator absent’ treatment. This decrease was accompanied with more divergent evolution also in the other life-history traits measured. In contrast, the replicate selection lines evolved in a more parallel manner within the ‘predator present’ treatment (Table 1, Fig. 2C&B). The populations within both treatments were originally generated from the same individual, and conditions were kept constant throughout the experiment. Therefore, all genetic variation within and between replicate selection lines arose initially from *de novo* mutations. Mutation accumulation can deteriorate bacterial traits randomly if the traits do not affect fitness in a given environment [25], [42], [55]–[59]. Our results are consistent with this, demonstrating that selection for bacterial traits was relaxed in the absence of predation. As a result, also virulence changed in some of the replicate selection lines probably due to random accumulation of mutations affecting unused virulence traits [25]–[26], [42]. While it has been demonstrated that bacterial virulence genes can evolve parallel between different hosts (i.e. replicate within-host populations) [60], our study shows that selection in the outside-host environment can also lead to parallel evolutionary changes in virulence.

In conclusion, our results show that virulence can decrease in an opportunistic bacterial pathogen if it is traded off with anti-predator adaptations, but also if randomly accumulating mutations impair virulence traits under relaxed selection. These results seem to contradict studies according to which protist predation selects for increased virulence in opportunistic bacteria [11]–[13], [61]. However, classifying opportunistic pathogens broadly to one category is an over-simplification [6]. Selection by protists (or by any other agent) is most likely highly case-specific and can thus lead to a positive or a negative correlation with virulence depending on the species and traits under selection (7). For example, amoebal predation can prepare an intracellular pathogen *Legionella* to resist human macrophages, whereas ciliate predation decreases *Serratia* virulence via a trade-off between prodigiosin and biofilm. Negative life-history correlations are likely to be especially important with environmentally transmitted bacteria that regularly encounter conflicting selection pressures in within-host and outside-host environments [8]. For example, two different variants of *Burkholderia ambifaria* cystic fibrosis isolates have superior fitness either in plant rhizosphere or the lungs of cystic fibrosis patients [62]. If these kind of trade-offs between pathogenic and environmental life-history strategies are common, they could partly explain why opportunistic bacteria found from the environment are seldom highly virulent, and how polymorphism in virulence traits is maintained in environmentally transmitted pathogen populations.

## Supporting Information

Table S1 Pairwise comparisons of host survival when injected with clones from predator absent or predator present treatments, with ancestor clone, or with water.
(PDF)

Table S2 Pairwise comparisons of host survival when injected with red and white clones from both predator absent and predator present treatments.
(PDF)
