# Supplementary material for: Life History Trade-Offs and Relaxed Selection Can Decrease Bacterial Virulence in Environmental Reservoirs
Source: PLoS One. 2012 Aug 24;7(8):e43801. doi: 10.1371/journal.pone.0043801 (PMC3427151; doi:10.1371/journal.pone.0043801)
Supplement: Table S1 — Pairwise comparisons of host survival when injected with clones from predator absent or predator present treatments, with ancestor clone, or with water. (PDF) [file pone.0043801.s001.pdf]

Treatment:	Predator absent		Predator present		Ancestor		Water	
	χ^2	p=	χ^2	p=	χ^2	p=	χ^2	p=
Predator absent			10.7	0.001	120.0	<0.001	41.0	<0.001
Predator present	10.7	0.001			175.1	<0.001	27.0	<0.001
Ancestor	120.0	<0.001	175.1	<0.001			81.2	<0.001
Water	41.0	<0.001	27.0	<0.001	81.2	<0.001		
