# Supplementary material for: Life History Trade-Offs and Relaxed Selection Can Decrease Bacterial Virulence in Environmental Reservoirs
Source: PLoS One. 2012 Aug 24;7(8):e43801. doi: 10.1371/journal.pone.0043801 (PMC3427151; doi:10.1371/journal.pone.0043801)
Supplement: Table S2 — Pairwise comparisons of host survival when injected with red and white clones from both predator absent and predator present treatments. (PDF) [file pone.0043801.s002.pdf]

**Predation & Prodigiosin:**	Predator absent, White		Predator present, White		Predator absent, Red		Predator present, Red	
	χ^2	p=	χ^2	p=	χ^2	p=	χ^2	p=
Predator absent, White			6.8	0.009	7.0	0.008	9.8	0.002
Predator present, White	6.8	0.009			37.5	<0.001	38.2	<0.001
Predator absent, Red	7.0	0.008	37.5	<0.001			0.8	0.367
Predator present, Red	9.8	0.002	38.2	<0.001	0.8	0.367		
